# Relationship between herd-level incidence rate of energy-related postpartum diseases, general risk factors and claw lesions in individual dairy cows recorded at maintenance claw trimming

**DOI:** 10.1186/1751-0147-55-55

**Published:** 2013-07-24

**Authors:** Christel Nielsen, Lena Stengärde, Christer Bergsten, Ulf Emanuelson

**Affiliations:** 1 Department of Clinical Sciences, Swedish University of Agricultural Sciences, P.O. Box 7054, SE-750 07, Uppsala, Sweden; 2 Department of Biosystems and Technology, Swedish University of Agricultural Sciences, P.O. Box 86, SE-230 53, Alnarp, Sweden

**Keywords:** Dairy cow, Energy-related metabolic diseases, Claw trimming, Claw lesions, Risk factors

## Abstract

**Background:**

Laminitis and energy-related postpartum diseases share several risk factors, indicating a common etiology. Thus, a herd’s incidence rate of energy-related postpartum diseases, such as displaced abomasum and clinical ketosis, might reflect the likelihood of cows to suffer from laminitis-related claw lesions. The aim of this study was to investigate the association between herd-level incidence rate of displaced abomasum and clinical ketosis, general risk factors, and claw lesions in individual cows recorded at maintenance claw trimming.

**Methods:**

The dataset consisted of 6773 trimmings, performed between 2004 and 2006 by professional trimmers, from 3607 Swedish Red and Swedish Holstein cows in 26 herds. The herds were classified as having a high, inconsistent-high or low incidence rate of energy-related postpartum diseases, based on the number of recorded cases of veterinary-diagnosed displaced abomasum and clinical ketosis in the Swedish national animal disease recording system during 2002 to 2006, and observations and interviews in connections with herd visits. Generalized linear mixed models were used to investigate the association between herd-level incidence rate of energy-related postpartum diseases and laminitis-related lesions including sole ulcer and sole hemorrhage; and hygiene-related lesions including interdigital dermatitis, digital dermatitis, heel-horn erosion, verrucose dermatitis, and interdigital hyperplasia; and absence of any claw lesion. Systematic effects, including first-order interactions, with *P* < 0.05 were included in the models. Herd classification was forced into the models, and a random effect of herd was included.

**Results:**

In comparison to herds with a high incidence rate of energy-related postpartum diseases, low-incidence herds showed a lower odds ratio (OR; 0.2) for laminitis-related lesions in cows trimmed during the summer months. Low-incidence herds also showed numerically lower OR estimates for laminitis-related lesions in all parity classes and a numerically lower OR for hygiene-related lesions. In addition, low-incidence herds showed tendencies towards a numerically higher OR for absence of any lesion, irrespective of trimming season or parity.

**Conclusions:**

Only a few statistically significant associations were found, but several tendencies pointed towards better claw health in herds with low as compared with high incidence rate of energy-related postpartum diseases.

## Background

Lameness has great ethical and economic importance, and is considered to be one of the most serious welfare problems in dairy cattle [1]. The major cause of lameness is claw lesions [2]. A prevalence study based on protocols from maintenance claw trimmings demonstrated that 72% of Swedish dairy cows had at least one claw lesion [3]. This figure is in agreement with the prevalence reported in contemporary Western European studies [4,5]. Lameness is associated with adverse production effects: reduced milk yield [6-8], reduced fertility [9-11], and increased risk of culling [12,13]. The cost of lameness has been estimated at €192 per first lactational incidence in Denmark [14] and of claw disorders (clinical and subclinical) at $75 per cow and year for the average farm in the Netherlands [15].

Claw lesions can be divided into laminitis and related horn lesions, infectious lesions, and lesions caused by excessive wear or trauma [16]. Risk factors vary between lesions and can be divided into systemic factors associated with calving and onset of lactation [17], managerial practices around calving [18], metabolic and environmental causes [19-21], and claw trimming routines [22-24]. Cow-related factors such as breed, parity, stage of lactation, and milk yield also play important roles in the development of claw lesions [8,20,25,26].

A major challenge in managing claw health in dairy herds is laminitis. Laminitis-related lesions in cattle are chronic in nature and the treatment given is often conservative, resulting in a long recovery period and exaggerated sensitivity to pain also 28 days after treatment [27]. In addition, calculations have shown that sole ulcer, a laminitis-related lesion, is associated with a substantially higher cost per case than other claw lesions [28], because of long recovery time and guarded prognosis [29].

Laminitis-related lesions are of multifactorial origin. Risk factors are often associated with management changes experienced by the transition cow [17,30,31]. For instance, intensive feeding and hard floor surfaces are proven causative factors of laminitis [4,17,18,24], and abrupt changes to these conditions in connection with calving further increase the risk of laminitis-related claw horn lesions [31,32].

Diets with a high proportion of rapidly fermentable carbohydrates and an insufficient quantity and quality of fiber increase the risk of laminitis [24,33-35], as well as for displaced abomasum [36,37] and subacute ruminal acidosis [38-40]. Furthermore, a negative energy balance has been identified as a predisposing factor for both laminitis and for metabolic and digestive problems occurring in the transition period [41]. Evidence thus suggests a common etiology, and so a herd’s incidence rate of energy-related postpartum diseases might reflect the likelihood of cows to suffer from laminitis-related claw lesions. In addition, energy-related postpartum diseases, e.g. displaced abomasum and clinical ketosis, *per se* have been reported to increase the odds ratio (OR) for feet and leg disorders [42].

The main aim of the present study was to investigate the relationship between the long-term herd-level incidence rate of displaced abomasum and clinical ketosis and the prevalence of claw lesions in individual cows recorded at maintenance claw trimming. In addition, also general risk factors for recorded claw lesions were investigated.

## Material and methods

### Herd selection

Herds with high or low long-term incidence rate of energy-related postpartum diseases and with records from maintenance claw trimmings were included in the study. Herd status with respect to incidence rate of these diseases was identified in a larger investigation of risk factors for energy-related postpartum disorders around calving in high-producing dairy cows [43]. Only herds with enrolment in the Swedish official milk-recording scheme (SOMRS), geographic location in one of four provinces of Sweden (Skåne, Halland, Västergötland, and Uppland), and a herd size of at least 50 cows were eligible for inclusion. Demographic information on herds and cows was retrieved from SOMRS. Herd size was defined as the average number of cow-years during 2004 to 2006.

### Identification of high and low incidence herds

For the purpose of the larger investigation, herds were classified as having a high or low long-term incidence rate of energy-related postpartum diseases based on recorded cases of veterinary treated displaced abomasum and clinical ketosis in the Swedish national animal disease recording system (NADRS). Cases of displaced abomasum included cows having displacement of the abomasum to the left or to the right with or without suspected torsion. Clinical ketosis was defined as cows in early lactation with inappetence, depressed general condition and reduced milk yield and tested positive for ketone bodies in milk, where the veterinarian found no other explanation for the symptoms. These diseases were chosen because they generally require veterinary treatment. In Sweden, it is mandatory to report all veterinary treatments to NADRS. Thus, this provided access to a reliable source of data. Other energy-related postpartum diseases such as hepatic lipidosis and diseases with a presumed metabolic component (e.g. retained placenta, udder edema, and subclinical rumen acidosis) would have been relevant to include in the study to avoid misclassification of herds, but these diseases are not always veterinary treated and therefore not included in NADRS in a consistent manner. Thus, displaced abomasum and clinical ketosis were considered marker diseases for the complex of energy-related postpartum diseases occurring in the transition period.

The incidence rate of displaced abomasum and clinical ketosis in the NADRS database during three consecutive 12-month periods (April to March, 2002 to 2005) were studied retrospectively to identify high or low incidence herds suitable for visits between January 2005 and June 2006. For each herd and period, the incidence rate was calculated as the number of cows with either of the two diagnoses in the NADRS database, divided by the number of cow-years during the period. Herds were divided into high-incidence and low-incidence herds by comparing their incidence rates to the 3^rd^ and 1^st^ quartiles for each of the three 12-month periods. Herds with an incidence rate above the 3^rd^ quartile during the last 12-month period and during at least one of the preceding two 12-month periods (0.04, 0.04, and 0.03 disease cases per cow-year, respectively) were considered high-incidence herds, while those with an incidence rate equal to the 1^st^ quartile (0 for all three periods) were considered low-incidence herds. After the herd visits, the retrospective herd classification was confirmed or revised according to up-to-date incidence rates of displaced abomasum and clinical ketosis based on observations of disease incidence in the herds, interviews with the farmers and on data in the NADRS database (i.e. from July to June, 2004 to 2005 or 2005 to 2006, in herds visited before or after October 2005, respectively). An incidence rate above the 3^rd^ quartile (0.04 disease cases per cow-year) qualified herds as having a high incidence rate of energy-related postpartum diseases at the time of the visit. An incidence rate equal to the 1^st^ quartile (0 disease cases per cow-year) and a maximum of 0.01 disease cases per cow-year qualified herds as having a low incidence rate of energy-related postpartum diseases at the time of the visit. Herds retained their classification if the initial classification was consistent with the revised one. If not, herds were classified as inconsistent-high if they were initially classified as high incidence herds but the classification at the herd visit was either low or intermediate. Likewise, herds were classified as inconsistent-low if they were initially classified as low incidence herds but the classification at the herd visit was either intermediate or high.

### Claw lesions

Data on maintenance claw trimmings, performed by professional claw trimmers between January 2004 and December 2006, was retrieved from SOMRS. Claw lesions were diagnosed by the trimmers, who had been trained to recognize the most commonly observed lesions [44]. Clinical and subclinical cases of sole ulcer (including ulceration and abscess of toe and white line), sole (white line) hemorrhage, double sole, fissure of the white line, dermatitis (interdigital and digital dermatitis), heel-horn erosion, verrucose dermatitis (wart growth), and interdigital hyperplasia (corns) were recorded ( [3]; Additional file 1). That means for example that a severe dermatitis is equal with digital dermatitis according to Döpfer [45], and a severe ulceration of the white line is equal with a white line abscess. The severities of sole ulcer, sole hemorrhage, dermatitis, and heel-horn erosion were recorded as 1 (affected) or 2 (severely affected). However, very few cases were scored as 2 in herds with a low incidence rate of energy-related postpartum diseases (range 0–5 depending on diagnosis), so severity of lesion was not considered in the analyses and the lesions were classified to be present or absent. However, even without classifying by severity, some diagnoses occurred in only small numbers among herds with a low incidence rate of energy-related postpartum diseases. Therefore, the diagnoses were merged into *laminitis*-*related lesions* including ulceration of sole, toe and white line, and sole hemorrhage, and *hygiene*-*related lesions* including dermatitis, heel-horn erosion, verrucose dermatitis, and interdigital hyperplasia.

### Dataset

Only one herd was classified as having an inconsistent-low incidence rate of energy-related postpartum diseases and was excluded from the study. After editing, the dataset consisted of 6773 trimming records collected from 3607 cows in 26 herds. The average herd size was 133 cows (standard deviation (SD) = 82). The average number of recorded trimmings per herd was 261 (SD = 255) and the average number of recorded trimmings per cow was 1.9 (SD = 1.2).

Cows were of the Swedish Holstein (SH; *n* = 2900) and Swedish Red (SR; *n* = 707) breeds and had an average yearly production of 9766 kg energy-corrected milk (( ECM (kg/day) = milk (kg/day) × [38.3 × fat (g/kg) + 24.2 × protein (g/kg) + 16.54 × lactose (g/kg) + 20.7]/3140)). They were housed in free stalls (*n* = 21) or tie stalls (*n* = 5) and were on mandatory pasture during the summer months. In 11 herds, cows were fed roughage and concentrates separately, while the remaining 15 herds used total mixed rations. Ten of the latter herds supplemented these diets with concentrates and the feeding was classified as partial mixed ration. In the free stalls concentrates were fed in computer feeding stations and in some herds at milking.

### Statistical methods

#### Clustering of observations

The structure of the dataset was hierarchical with four levels: region, herd, cow, and trimming. We were not interested in including any predictors at the regional level, and so region was omitted from the analyses. Clustering of cows within herds was accounted for by including herd as a random effect in the models. About half of the cows had multiple trimmings, and trimmings were therefore partially clustered within cows. Unless accounted for, this partial clustering of trimmings could potentially result in underestimated between-cow variance, because for cows with only one observation, part of this variance would be attributed to the error term. This would, in turn, give rise to biased estimates of herd-level independent variables and too small standard errors.

To investigate the importance of within-cow clustering of trimmings, the intra-class correlation coefficient was calculated. All observations in the dataset were used to fit a linear mixed model with absence of any claw lesion as the dependent variable. Although the dependent variable was a binary trait, the frequency of absence of any lesion was 52%, and so the approximation implied by the choice of model was judged as being reasonably correct. The model corrected for herd classification (i.e. high-incidence and low-incidence herds), trimming season, breed, parity, lactation stage, and interactions of herd classification and trimming season and herd classification and parity (same effects as in the final model as explained later), and included herd and cow as random effects. The intra-class correlation coefficient of cows within herd was 0.21 and that of trimmings within cow was 0.34, indicating that clustering of observations might affect the standard errors of the estimates. In the model for laminitis-related lesion, the intra-class correlation coefficient of cows within herd was 0.20 and that of trimmings within cows was 0.28. In the model for hygiene-related lesions, the intra-class correlation coefficient of cows within herd was 0.16 and that of trimmings within cows was 0.29.

The practical impact of clustering was investigated by fitting three linear mixed models, including random effects of herd and cow, random effect of herd, and no random effect, respectively, with absence of any lesion as the dependent variable, to a subset of the data including only observations from cows with multiple trimmings (4933 records). The least-squares means and standard errors obtained when herd and cow, as compared with only herd, were included as random effects in the model showed only very minor differences. The model without any random effect did, however, yield substantially lower standard errors and affected least-squares means. This suggested that clustering within herds was extremely important, whereas clustering within cows was less important. Based on these findings, the final analyses were performed on the complete dataset, using models accounting only for clustering of observations within herds. The same procedure was repeated to assess the practical implications of the intra-class correlations in the models for laminitis-related and hygiene-related lesions, and the results and conclusions were similar.

#### Statistical analyses

The effects of herd-level incidence rate of energy-related postpartum diseases on claw health in individual cows at maintenance claw trimmings were estimated using generalized linear mixed models, specified as having a binomial distribution and using a logit link function, in PROC GLIMMIX in SAS (Version 9.2, SAS Institute Inc., Cary, NC, USA). Trimming records from 11 inconsistent-high herds were included in the analyses to improve the accuracy of estimates of fixed effects. Dependent variables were laminitis-related lesions, hygiene-related lesions, and absence of any lesion (1 = present; 0 = absent). Models were built using manual backward elimination. Predictor variables at herd level, i.e. herd classification (high, inconsistent-high or low); housing system (tie stalls or free stalls); feeding regimen (total mixed ration, partial mixed ration, or component ration); average herd size; and average yearly milk production per cow, and cow level, i.e. breed (SH or SR); parity (1, 2, or ≥ 3); lactation stage (≤ 2 months, > 2 to 5 months, > 5 to 10 months, or > 10 months); and trimming season (January to April, May to August, or September to December), were offered to the model. Predictor variables, including first-order interactions, with associations significant at the *P* < 0.05 level were included in the final models. Herd classification was forced into all models, and a random herd effect was included. Models were validated by the Hosmer-Lemeshow goodness-of-fit test, which provided no evidence of lack of fit of the models for laminitis-related lesions (*P* = 0.11) and absence of any lesion (*P* = 0.065), but suggested that the fit of the model for hygiene-related lesions was somewhat compromised (*P* = 0.045). The results of the latter should thus be interpreted with caution.

All animal handling and sampling for the study was approved by the local ethics committee in Uppsala, Sweden.

## Results

### Descriptive statistics

Table 1 gives the distribution of herds, cows, and trimming records over herd classifications. Four herds were defined as having a low incidence rate of energy-related postpartum diseases, and 7% of the trimmings were performed in these herds. The average number of trimmings per cow was higher in herds with a high incidence rate of energy-related postpartum diseases.

**Table 1:** **Number and distribution of herds**, **cows**, **and trimming records according to herd classifications of incidence rates of energy related postpartum diseases**

	**High**				**Inconsistent**-**high**				**Low**			
	***n***	**Mean**^**1**^	**S.D.**^**2**^	**Range**	***n***	**Mean**	**S.D.**	**Range**	***n***	**Mean**	**S.D.**	**Range**
Herds	11	-		-	11	-		-	4	-		-
Cows	1530	139	88	49-283	1713	156	125	61-437	364	91	67	36-175
Trimmings	2845	1.9	1.3	1-7	3451	2.0	1.1	1-6	477	1.3	0.5	1-2

^1^Mean *n* per unit at next-higher level.

^2^Standard deviation.

The prevalence of all lesions except sole ulcer was substantially higher in herds with a high incidence rate of energy-related postpartum diseases (Table 2). In these herds, at least one lesion was detected at 57% of the trimmings. The corresponding figure in herds with a low incidence rate of energy-related postpartum diseases was 39%. The percentage of trimmings where cows suffered from both laminitis-related and hygiene-related lesions was 24%, 8% and 10%, respectively, in herds with high, inconsistent-high and low incidence rate of energy-related postpartum diseases.

**Table 2:** **Prevalence of claw lesions at trimmings in herds with different incidence rates of energy**-**related postpartum diseases**

	**High**		**Inconsistent**-**high**		**Low**	
	***n***	**Prevalence (%)**	***n***	**Prevalence (%)**	***n***	**Prevalence (%)**
*Laminitis*-*associated lesion*^1^	1141	40	772	22	125	26
Sole ulcer	157	6	146	4	27	6
Sole haemorrhage	1071	38	674	20	110	23
*Hygiene*-*related lesion*	1160	41	931	27	108	23
Digital dermatitis	183	6	247	7	6	1
Heel-horn erosion	839	29	521	15	74	16
Other lesions^2^	446	16	283	8	42	9
*Absence of any lesion*	1229	43	2032	59	291	61

^1^Observations of individual lesions do not sum to the number of lesions in disease complexes because cows could have multiple lesions per trimming.

^2^Other lesions include interdigital dermatitis, verrucose dermatitis, and interdigital hyperplasia.

### Laminitis-related lesions

The final model of risk factors associated with presence of laminitis-related lesions is presented in Table 3. A lower OR during the summer months was observed in herds with low as compared with high incidence rate of energy-related postpartum diseases (OR = 0.15, 95% CI: 0.02 – 0.95). In all parity classes, there were numerically lower OR estimates in herds with a low incidence rate of energy-related postpartum diseases (parity 1: OR = 0.85, 95% CI: 0.18 – 3.96; parity 2: OR = 0.49, 95% CI: 0.10 – 2.33; parity ≥ 3: OR = 0.48, 95% CI: 0.10 – 2.25). The OR was lower in cows trimmed during September to December as compared with cows trimmed during January to April (high-incidence herds: OR = 0.47, 95% CI: 0.34 – 0.64; inconsistent-high-incidence herds: OR = 0.62, 95% CI: 0.47 – 0.82). No clear trend was observed for the impact of parity in different herd classifications, although cows of third and higher parities in herds with a high incidence rate of energy-related postpartum diseases had an OR of 1.61 (95% CI: 1.27 – 2.04) as compared with primiparous cows. Primiparous cows were more susceptible than multiparous cows during the first half of lactation, whereas the relationship was the opposite after the sixth lactation month. Swedish Red cows were less likely than Swedish Holstein cows to be diagnosed with laminitis-related lesions during the housing season (September to December: OR = 0.34, 95% CI: 0.24 – 0.49; January to April: OR = 0.54, 95% CI: 0.38 – 0.76), while there was no difference during the pasture season (May to August). Irrespective of parity, the OR was highest during the third to fifth month of lactation.

**Table 3:** **Results from the mixed**^**1 **^**logistic regression model of risk factors associated with presence of laminitis**-**associated claw lesions**

	**Coefficient**	**CI**^**2**^	**OR**^**3**^	***p-*value**
*Herd classification*^*4*^ × *trimming season*				<0.001
High × Jan-April	Ref		1.00	
Low × Jan-April	−0.15	−1.69 − 1.39	0.86	
Inconsistent-high × Jan-April	−0.91	−2.01 − 0.18	0.40	
High × May-Aug	−0.03	−0.35 − 0.30	0.97	
Low × May-Aug	−1.91	−3.72 − −0.09	0.15	
Inconsistent-high × May-Aug	−0.55	−1.66 − 0.56	0.58	
High × Sept-Dec	−0.76	−1.08 − −0.45	0.47	
Low × Sept-Dec	−0.35	−1.85 − 1.16	0.71	
Inconsistent-high × Sept-Dec	−1.39	−2.49 − −0.29	0.25	
*Herd classification* × *parity*				0.018
High × 1	Ref		1.00	
Low × 1	−0.17	−1.71 − 1.38	0.85	
Inconsistent-high × 1	−0.39	−1.47 − 0.70	0.68	
High × 2	0.09	−0.17 − 0.35	1.09	
Low × 2	−0.62	−2.17 − 0.93	0.54	
Inconsistent-high × 2	−0.77	−1.87 − 0.32	0.46	
High × ≥3	0.48	0.24 − 0.71	1.61	
Low × ≥3	−0.26	−1.80 − 1.29	0.77	
Inconsistent-high × ≥3	−0.34	−1.43 − 0.75	0.71	
*Breed* × *trimming season*				<0.001
Swedish Holstein × Jan-April	Ref		1.00	
Swedish Red × Jan-April	−0.62	−0.96 − −0.27	0.54	
Swedish Holstein × May-Aug	−0.76	−1.23 − −0.29	0.47	
Swedish Red × May-Aug	−0.80	−1.34 − −0.27	0.45	
Swedish Holstein × Sept-Dec	−0.25	−0.48 − −0.02	0.78	
Swedish Red × Sept-Dec	−1.32	−1.73 − −0.92	0.27	
*Parity* × *lactation stage*				<0.001
1 × ≤2 months	Ref		1.00	
1 × >2 to 5 months	1.37	1.09 − 1.65	3.94	
1 × >5 to 10 months	−0.34	−0.62 − −0.06	0.71	
1 × >10 months	−0.78	−1.10 − −0.46	0.46	
2 × ≤2 months	−0.68	−1.10 − −0.27	0.51	
2 × >2 to 5 months	0.64	0.28 − 1.00	1.90	
2 × >5 to 10 months	−0.31	−0.66 − 0.03	0.73	
2 × >10 months	−0.39	−0.79 − 0.001	0.68	
≥3 × ≤2 months	−0.12	−0.50 − 0.25	0.88	
≥3 × >2 to 5 months	0.67	0.34 − 1.01	1.96	
≥3 × >5 to 10 months	0.31	−0.02 − 0.64	1.36	
≥3 × >10 months	−0.03	−0.39 − 0.34	0.97	

^1^Herd was included as a random effect.

^2^95% confidence interval.

^3^Odds ratio.

^4^Herds classified according to incidence rates of energy related postpartum diseases.

### Hygiene-related lesions

The final model of risk factors associated with the presence of hygiene-related lesions is presented in Table 4. Herd classification was not significant, although there was a numerically lower OR (0.39, 95% CI: 0.09 – 1.69) in herds with low as compared with high incidence rate of energy-related postpartum diseases. The OR was lowest during the summer months (May to August: OR = 0.33, 95% CI: 0.27 – 0.41) and highest in cows trimmed during January to April. Irrespective of when in lactation cows were trimmed, the OR increased with increasing parity. In primiparous cows, the OR generally increased as lactation progressed.

**Table 4:** **Results from the mixed**^**1 **^**logistic regression model of risk factors associated with presence of hygiene**-**related claw lesions**

	**Coefficient**	**CI**^**2**^	**OR**^**3**^	***p-*value**
*Herd classification*^*4*^				0.24
High	Ref		1.00	
Low	−0.94	−2.40 – 0.53	0.39	
Inconsistent-high	−0.77	−1.83 - 0.28	0.46	
*Trimming season*				<0.001
Jan-April	Ref		1.00	
May-Aug	−1.10	−1.31 – -0.89	0.33	
Sept-Dec	−0.64	−0.78 – -0.50	0.53	
*Parity* × *lactation stage*				<0.001
1 × ≤2 months	Ref		1.00	
1 × >2 to 5 months	0.33	0.03 – 0.63	1.39	
1 × >5 to 10 months	0.72	0.45 – 1.00	2.06	
1 × >10 months	0.65	0.36 – 0.95	1.92	
2 × ≤2 months	0.97	0.63 – 1.30	2.63	
2 × >2 to 5 months	0.97	0.66 – 1.28	2.64	
2 × >5 to 10 months	0.97	0.68 – 1.26	2.63	
2 × >10 months	0.98	0.66 – 1.31	2.68	
≥3 × ≤2 months	1.35	1.03 – 1.67	3.86	
≥3 × >2 to 5 months	1.25	0.95 – 1.54	3.48	
≥3 × >5 to 10 months	1.21	0.92 – 1.49	3.34	
≥3 × >10 months	1.16	0.84 – 1.48	3.20	

^1^Herd was included as a random effect.

^2^95% confidence interval.

^3^Odds ratio.

^4^Herds classified according to incidence rates of energy related postpartum diseases.

### Absence of any lesion

The final model of factors associated with absence of any claw lesion is presented in Table 5. There were numeric trends towards a higher OR in herds with low as compared with high incidence rate of energy-related postpartum diseases, irrespective of trimming season or parity. The OR was lowest in cows trimmed during January to April (although non-significant as compared with cows trimmed in September to December in herds with low incidence rate of energy-related postpartum diseases). In herds with high incidence rate of energy-related postpartum diseases, the OR decreased with increasing parity (parity 2: OR = 0.69, 95% CI: 0.54 – 0.87; parity ≥ 3: OR = 0.43, 95% CI: 0.34 – 0.54). Swedish Red cows were more likely than Swedish Holstein cows to have good claw health at the time of trimming (OR = 1.57, 95% CI: 1.30 – 1.89) and the OR was significantly lower for trimmings during the third to tenth month of lactation (third to fifth month: OR = 0.46, 95% CI: 0.38 – 0.54; sixth to tenth month: OR = 0.80, 95% CI: 0.68 – 0.94).

**Table 5:** **Results from the mixed**^**1 **^**logistic regression model of risk factors associated with absence of any claw lesion**

	**Coefficient**	**CI**^**2**^	**OR**^**3**^	***p-*value**
*Herd classification*^*4*^ × *trimming season*				0.007
High × Jan-April	Ref		1.00	
Low × Jan-April	0.96	−0.62 – 2.53	2.60	
Inconsistent-high × Jan-April	1.15	0.03 – 2.27	3.16	
High × May-Aug	0.80	0.46 – 1.14	2.23	
Low × May-Aug	2.14	0.52 – 3.77	8.52	
Inconsistent-high × May-Aug	1.49	0.37 – 2.61	4.43	
High × Sept-Dec	0.74	0.50 – 0.98	2.10	
Low × Sept-Dec	1.21	−0.32 – 2.74	3.34	
Inconsistent-high × Sept-Dec	1.62	0.51 – 2.73	5.05	
*Herd classification* × *parity*				0.001
High × 1	Ref		1.00	
Low × 1	0.54	−1.01 – 2.09	1.71	
Inconsistent-high × 1	0.59	−0.52 – 1.70	1.81	
High × 2	−0.37	−0.61 – -0.14	0.69	
Low × 2	0.62	−0.93 – 2.17	1.86	
Inconsistent-high × 2	0.57	−0.54 – 1.68	1.77	
High × ≥3	−0.85	−1.08 – -0.62	0.43	
Low × ≥3	0.37	−1.18 – 1.93	1.45	
Inconsistent-high × ≥3	0.32	−0.79 – 1.43	1.38	
*Breed*				<0.001
Swedish Holstein	Ref		1.00	
Swedish Red	0.45	0.26 – 0.64	1.57	
*Lactation stage*				<0.001
≤2 months	Ref		1.00	
>2 to 5 months	−0.78	−0.96 – -0.61	0.46	
>5 to 10 months	−0.23	−0.39 – -0.07	0.80	
>10 months	0.01	−0.17 – 0.19	1.01	

^1^Herd was included as a random effect.

^2^95% confidence interval.

^3^Odds ratio.

^4^Herds classified according to incidence rates of energy related postpartum diseases.

## Discussion

### Associations between energy-related postpartum **disease**s and claw lesions

A significantly lower OR for laminitis-related lesions in herds with low as compared with high incidence rate of energy-related postpartum diseases was found in cows trimmed during the summer months. As the claw horn lesions reflects an injury of the claw horn that occurred at least 2 months earlier and which may remain for months after, depending on claw horn wear, it is likely that this difference reflects the situation from the housing period [46]. Evidence suggests a common etiology between energy-related postpartum diseases and laminitis-related lesion. Overfeeding and overconditioning during the dry period, makes the cow more prone to metabolic disease such as displaced abomasum and ketosis [47,48]. Overconditioning together with weakened suspensory structures around calving [32] may also increase traumatic influence and risk for sole horn lesions to occur [49]. A negative energy balance in dairy cows is common in the transition period, and is partially caused by reduced dry matter and/or energy intake during this period [50,51]. On the other hand, lameness leads to reduced feeding times and the feeding behavior [52,53] causing negative energy balance, which may be a risk factor for displaced abomasum and clinical ketosis. Feeding factors associated with displaced abomasum and ketosis includes component feeding, feeding maize and a low fiber content [37,54,55]. These factors have also been associated with laminitis [24,35].

There is still a substantial lack of knowledge whether the relationships between the energy-related postpartum diseases, laminitis-related claw horn lesions, rumen acidosis and other parameters are really causative.

Numerically lower OR estimates for laminitis-related lesions in herds with low as compared with high incidence rate of energy-related postpartum diseases were also found in cows trimmed during January to April and in all parities. Likewise, the OR for hygiene-related lesions was numerically lower, although not statistically significant because of large standard errors, and there were numerically higher OR estimates for absence of any lesion in low-incidence herds, irrespective of trimming season or parity. The low number of lesions recorded in herds with a low incidence rate of energy-related postpartum diseases, and the few herds with low incidence rate, resulted in low power of the analyses. Assuming a power of 80% and 95% confidence, 152 lesions in each herd classification would have been required to detect a significant difference if the prevalence of lesions was 0.4 and 0.25, respectively [56]. Given that the dataset contained 125 laminitis-related lesions and 108 hygiene-related lesions in herds with a low incidence rate of energy-related postpartum diseases, the analyses may have failed to detect biological associations that were in fact present.

Another explanation of less obvious relationships in this study is the influence of trauma in the etiology of laminitis-related claw lesions [31]. In herds with poor housing facilities, where cows are challenged by prolonged standing times, for hard and abrasive floors, and by poor cow comfort [57] the risk for laminitis related lesions will be high and the metabolic influence may be less accentuated. Rubber mats will reduce the traumatic influence on claw horn lesions [58,59]. Most Swedish tie stalls are equipped with rubber mats while there is a large variation in free stall herds. Specific housing conditions, other than tie or free stalls, were not regarded in the present study and would have required inclusion of more herds to be able to analyze.

### Associations between claw lesions and other risk factors

#### Season of claw trimming

Housed cattle are at increased risk of developing infectious, hygiene-related claw lesions, for instance due to fecal contamination [60] or too hard floors [18,61]. Swedish legislation states that cows must be on pasture during the summer months; varying between 2 and 4 months depending on location (latitude, ie. growing season). The lower OR for hygiene-related lesions in cows trimmed during the grazing season found in the present study imply a curative effect due to improved hygiene and increased exercise, as suggested by Manske et al. [3]. Indeed, studies have reported that hygiene-related lesions do heal spontaneously, and it is less likely to get new such lesions at pasture [62,63].

#### Parity and lactation stage

Primiparous cows has been shown to be more prone to laminitis-related lesions than multiparous cows [22,61,64], which was confirmed by the present study. Primiparous cows had a higher OR for laminitis-related lesions during the first half of lactation, stressing the importance of the metabolic and environmental changes taking place around calving in the etiology of laminitis. Irrespective of parity, the OR for laminitis-related lesions was highest in the third to fifth month of lactation, supporting the findings of others [46,64,65]. Given the growth rate of sole horn, it can take up to three months before any visible signs of hemorrhage appear [64,66]. Laminitis-related lesions developing around calving are, therefore, often not detected until months later. Again, the results confirm the transition period as critical for the development of laminitis.

#### Breed

Swedish Red cows had better claw health than Swedish Holstein cows, in accordance with the results of Bergsten [61,67] and Manske et al. [22]. Higher susceptibility of Holstein cows to claw lesions and lameness as compared with cows of other breeds has also been reported in other countries [46,68,69]. The higher yield level of Holstein cows has been proposed as an explanation. However, even after adjusting for yield level in the analyses, Barker et al. [69] reported a significant association between lameness prevalence and breed, indicating that factors other than yield level are responsible for the poorer claw health in Holstein cows. Swedish Holsteins have been associated with a higher risk of displaced abomasum [55] as well as lameness.

In Sweden, the breeding goal of Swedish Red cows has traditionally put more emphasis on functional traits, such as disease resistance and longevity, than the breeding goal of Holstein cows. This offers an explanation for their lower OR for laminitis-related lesions; the more robust Swedish Red cow might be better able to cope with management changes and hard floors during the housing season.

#### Housing system

Although housing system is commonly reported as an important factor affecting the prevalence of claw lesions [70-72], it was not a significant predictor in any of the models. However, only five of the herds were housed in tie stalls, with only one of these having a high incidence rate of energy-related postpartum diseases and two having a low incidence rate. The absence of an effect of housing system might, consequently, be an artifact of the data structure. An alternative explanation could be that management routines in individual herds (absorbed in the random-herd effect) were more important than the housing system.

### Study design

#### Herd selection

As herds were selected and studied primarily for an investigation on energy-related postpartum diseases [43], emphasis was more on details in diagnosing them with expense on the number of herds. With a higher number of herds and including more parameters on management system it would have allowed a more complete analysis of non-metabolic parameters.

#### Frequency of claw trimming

Cows in herds with a high incidence rate of energy-related postpartum diseases underwent more frequent trimmings than those in herds with a low incidence rate of energy-related postpartum diseases (Table 1). This may indicate poorer claw health in high-incidence herds, forcing trimmings to occur more often [73]. It is, however, more likely that the frequent trimming actually reduced the prevalence as well as the incidence of new lesions. For instance, Manske et al. [22] found that an additional yearly trimming made in the autumn, as compared with only one trimming in the spring, reduced the prevalence of lame cows and laminitis-related lesions, as well as the need for acute trimmings in between maintenance claw trimmings. Thus, it is conceivable that the herds with a high incidence rate of energy-related postpartum diseases showed fewer lesions than what would have been observed with less frequent trimming. Otherwise, low incidence herds with fewer trimmings should have had more claw lesions, which was not the case in this study [3].

#### Claw trimmer

Unfortunately, the dataset did not contain information on the identity of trimmers, and so we could not control for the skill of the individual recording the diagnosis in the analyses. However, the inter-rater agreement among Swedish trimmers for claw lesions assessed using ordinal scores was acceptable to good [74], and the propensity of trimmers to record severe lesions, especially sole ulcer, was high [44]. Furthermore, the potential bias introduced by not including the identity of trimmer in the analyses would most likely be non-differential, because herd classification and trimmer was probably not systematically related and results would thus err towards the null hypothesis of no differences between herd classifications.

#### Confounding

Confounding bias is always a concern, especially in observational studies where information may not always be available to control for such bias. In this study it is possible that housing and management factors, such as overcrowding and floor surface, could be associated both with the risk factor under study and the outcome. However, this information was not collected and could therefore not be included in the multivariable model.

## Conclusion

Only a few statistically significant associations were found, but several tendencies pointed towards better claw health in herds with low as compared with high incidence rate of energy-related postpartum diseases. More knowledge is needed to clarify causative mechanisms and the possibly common metabolic etiology behind energy-related postpartum diseases and laminitis-related claw horn lesions.

## Competing interests

The authors declare that they have no competing interests.

## Authors’ contributions

CN carried out the statistical analyses, interpreted the results and drafted the manuscript. LS participated in the design of the study, collected the data and helped in the interpretation of the results and to draft the manuscript. CB participated in the interpretation of the results and helped to draft the manuscript. UE participated in the design of the study, the statistical analyses and the coordination, and helped to draft the manuscript. All authors read and approved the final manuscript.

## Supplementary Material

Additional file 1 Claw lesion color atlas. Click here for file
