# Supplementary material for: Relationship between herd-level incidence rate of energy-related postpartum diseases, general risk factors and claw lesions in individual dairy cows recorded at maintenance claw trimming
Source: Acta Vet Scand. 2013 Jul 24;55(1):55. doi: 10.1186/1751-0147-55-55 (PMC3737044; doi:10.1186/1751-0147-55-55)
Supplement: Additional file 1 — Claw lesion color atlas. [file 1751-0147-55-55-S1.pdf]

CLAW LESION COLOR ATLAS

DOUBLE SOLE (DS)

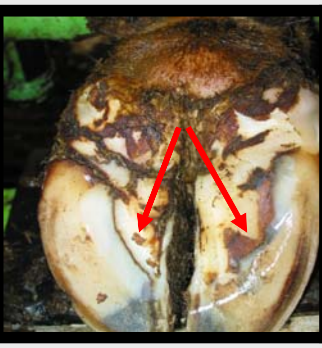

WL FISSURE (WF)

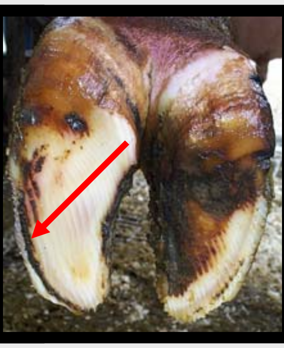

ABSCESS WL (WA)

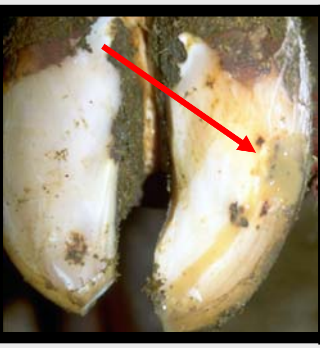

ABSCESS SOLE/TOE

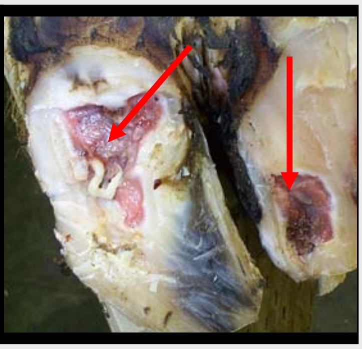

DIGITAL DERMATITIS (DD)

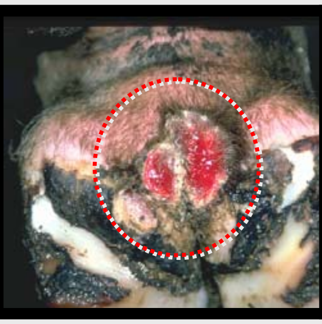

WART (PD)

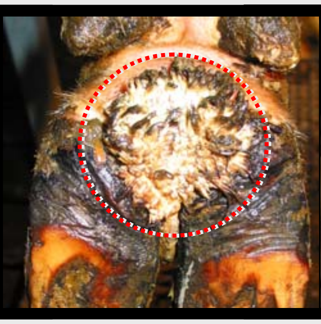

INT. HYPERPLASIA (IH)

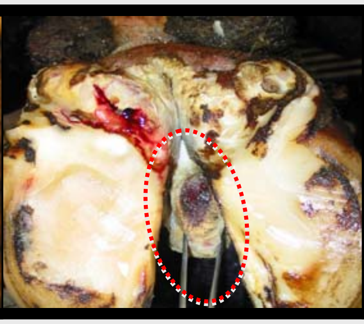

FOOT ROT (IP)

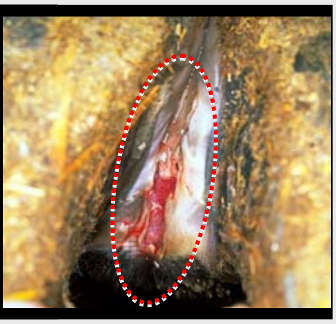

Abnormal Conformation

ASYMMETRY (AC)

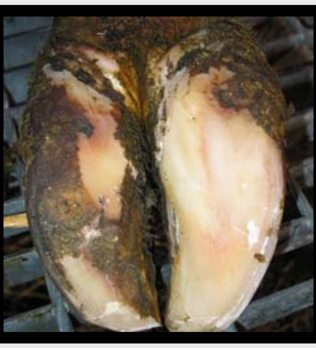

OVERGROWTH (OC)

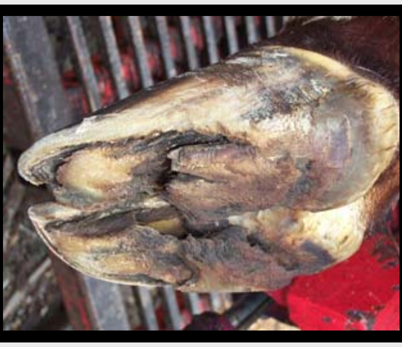

CORKSCREW (CC)

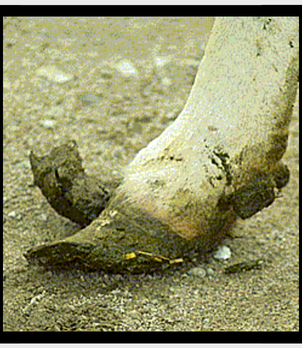

WEAK PASTERN (WP)

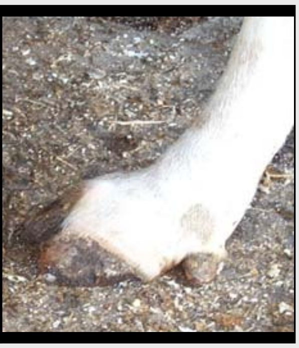

Locomotion

Mild lameness/stiffness (/)

STANDING

WALKING

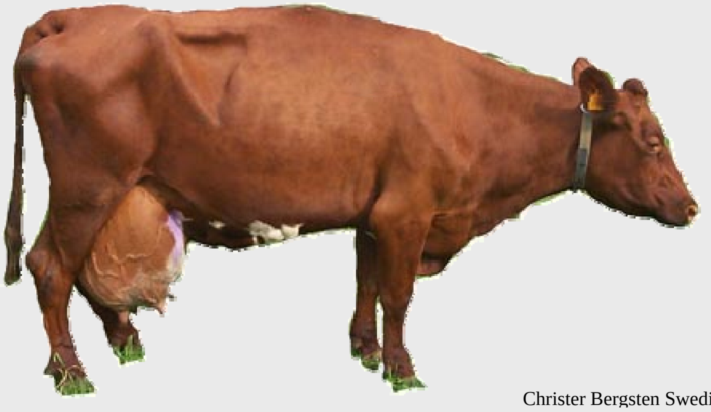

Moderate /severe lameness (X)

STANDING

WALKING

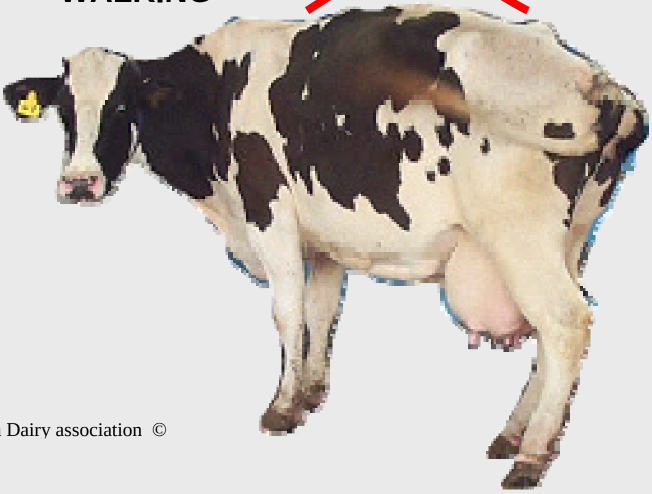

Christer Bergsten Swedish Dairy association ©

CLAW LESION COLOR ATLAS

MODERATE/SUPERFICIAL (/) SEVERE/PROFOUND (X)

SOLE HAEMORRHAGE (SH)

Haemorrhagic discoloration of the sole horn and/or white line. Diffuse symptom of (subclinical) laminitis 2-3 months after calving. Hard floors and intensive unbalanced feeding associated with poor rumination are important risk factors. Normally heals spontaneously but can develop to more severe lesions as ulceration of sole/toe/white line and/or double sole/white line lesions

Treatment and prevention

Trimming, balancing the outer and inner claws, and dishing out to unload the ulcer area is recommended. Rubber mats where the cows are walking and standing and optimized cow comfort are measures to prevent and reduce the risk for complications.

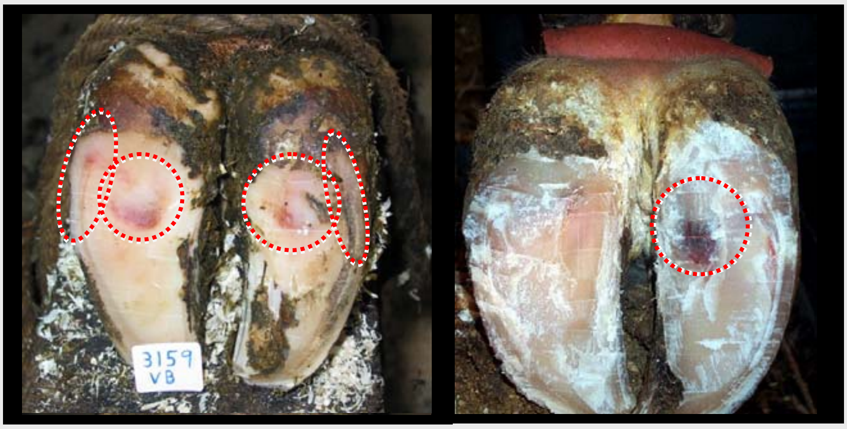

SOLE ULCER (SU)

Open wound (ulceration) of the sole, toe or white line often complicated by a deeper infection and or inflammation. Common cause of lameness. The ulcer is caused by compression of the corium between the claw bone and sole. Laminitis and trauma predisposes to or causes ulcers.

Treatment and prevention

Trimming and balancing of the outer and inner claws and dishing-out of the sole to unload the ulcer area. Blocking of the healthy claw to reduce pain and improve healing. Balanced diets, proper feeding, rubber mats where the cows are walking and standing, and optimized cow comfort prevent the condition.

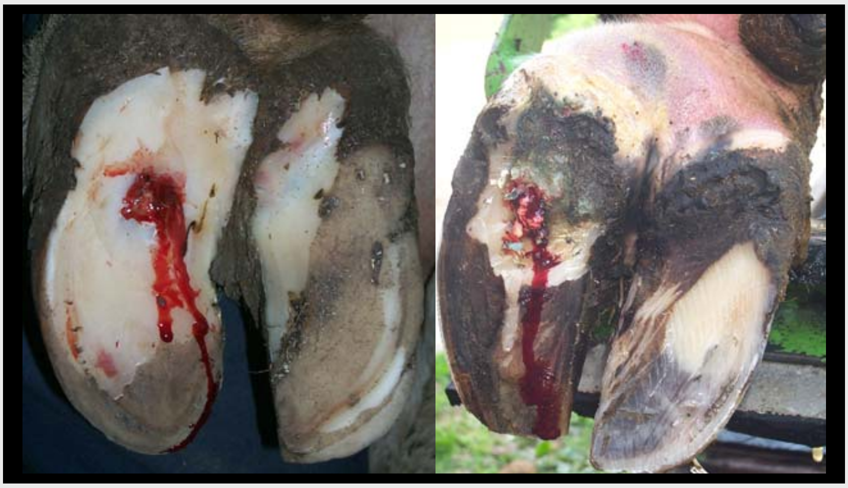

DERMATITIS (ID, DD, PDD)

Inflammation of the digital and/or interdigital skin with ulcerations or crusts. Mild/early stages do not cause lameness and are often neglected if feet are not cleansed. The dermatitis can progress to more severe, painful lesions (DD) causing severe lameness. Most often hygiene related herd problem that can spread from animal to animal and from herd to herd if biosecurity precautions are not undertaken.

Treatment and prevention

Mild to moderate dermatitis heals spontaneously with improved hygiene of stalls and feet. Proper foot bathing cleanses and disinfects feet and thus reduces the symptoms and controls spreading.

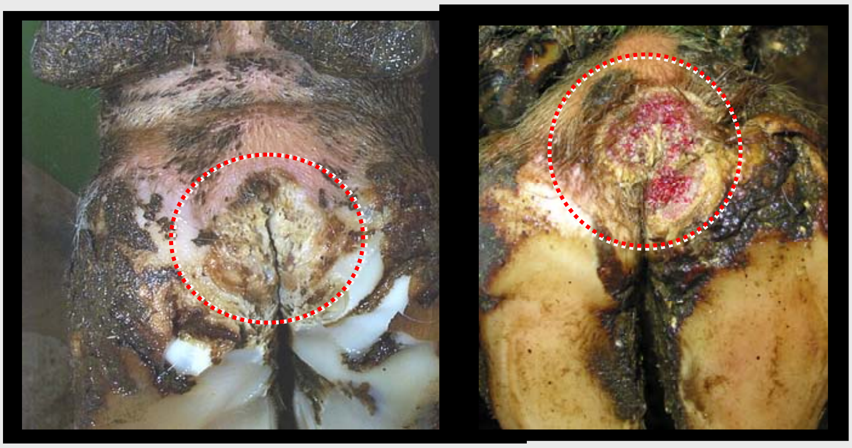

HEEL HORN EROSION (HE)

V-shaped erosions and clefts in the bulbs or circular craters that can undermine the heels. Most often double-sided and more common in the rear feet. Associated with dermatitis. Deeper erosions progressing to the corium may cause lameness.

Treatment and prevention

Trimming away diseased horn and treatment of dermatitis with antibacterial compounds locally. Foot bathing can prevent herd problems but most important to improve environment and foot hygiene.

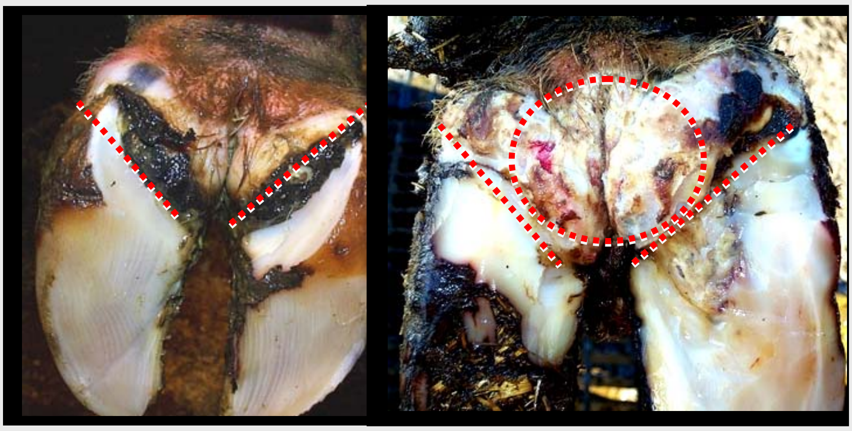

Christer Bergsten Swedish Dairy association ©
